# Implementing sensor technology applications for workplace health promotion: a needs assessment among workers with physically demanding work

**DOI:** 10.1186/s12889-019-7364-2

**Published:** 2019-08-14

**Authors:** Sander Mathijn Spook, Wendy Koolhaas, Ute Bültmann, Sandra Brouwer

**Affiliations:** Department of Health Sciences, Community and Occupational Medicine, University of Groningen, University Medical Center Groningen, Antonius Deusinglaan 1, P.O. Box 196, FA10, room 620, 9700 AD Groningen, the Netherlands

**Keywords:** Needs assessment, Focus groups, Sensor technology applications, Workplace health promotion, Physically demanding work, Work exposures, Occupational hazards, Sustainable employability, Ageing workforce

## Abstract

**Background:**

Workers with physically demanding work may be at risk for injury, illness or other adverse health outcomes due to exposure to different occupational hazards, especially at higher age. Sensor technology applications may be useful in the workplace to unobtrusively measure and monitor work exposures and provide workers with real-time feedback or access to data on demand. Many aspects might impede the implementation of sensor technology applications in the workplace, which should be taken into consideration for a successful implementation. Moreover, needs and preferences of workers regarding the use of sensor technology applications during work performance need to be identified. Therefore, the aim of this study was to identify worker needs and preferences regarding the use of sensor technology applications in the workplace.

**Methods:**

Four on-site focus group sessions were conducted in four different companies among workers with physically demanding work (*n* = 30). Semi-structured interview schedules were used to identify which work exposures should be measured, by which kind of sensor technology applications, under which (pre)conditions, how to motivate long-term use of sensor technology applications, and which type of feedback is preferred. For data analysis, a content-analysis with an inductive approach was performed.

**Results:**

Participants mentioned that they want to use wearable sensor technology applications to measure and monitor physical job demands, occupational heat stress, noise and fatigue. Factors associated with quality, comfort and perceived ease of use were identified as potential barriers for implementation in the workplace. Long-term motivation was attributed to the ability to manage and monitor work exposures, positive feedback and data ownership. Participants indicated a need to both receive real-time feedback and access to data on demand.

**Conclusions:**

Sensor technology applications may support workers with physically demanding work to measure and monitor their work exposures. Potential barriers for implementation such as privacy aspects and quality, comfort and perceived ease of use of sensor technology applications need to be well considered to ensure successful implementation of sensor technology applications in the workplace.

**Electronic supplementary material:**

The online version of this article (10.1186/s12889-019-7364-2) contains supplementary material, which is available to authorized users.

## Background

Workers with physically demanding work may be exposed to various occupational hazards when performing their daily work activities. During the past decades, many studies have shown that exposures such as pushing, pulling and heavy lifting [1–3], bending and twisting [1], adopting awkward postures [2, 4], long working hours [5–7], shift work [8], job control [9, 10] and exposure to occupational noise [11] and hazardous substances and fumes [12, 13] are linked with occupational health outcomes, such as work-related musculoskeletal disorders [2, 4, 10], hearing loss [11, 14], cancer [12] or cardiovascular disease [5, 6, 11, 12]. Certain occupational hazards impose increased risks for occupational injuries, illnesses or other adverse health outcomes which can result in unsafe work practices [14] and productivity loss [15]. When workers are affected by illness or injuries, this may result into (long-term) sickness absence [1, 3, 8, 9, 13], work disability [16] and premature exit from the labour market [3, 17–20]. Especially ageing workers in physically demanding jobs are vulnerable, as performing physically demanding work for many years may take a significant toll on the body [1] while the functional capacity to meet existing work demands declines with increased age [21]. Measuring and monitoring work exposures with sensor technology applications may help workers to prevent adverse health effects and assist employers in promoting workplace health. To date, it is unclear whether workers are actually willing to use sensor technology applications in the workplace.

So far, sensor technology applications have been implemented sporadically in the workplace. Work exposures and health consequences such as adopted work posture [22] or physical fatigue [23] can be unobtrusively monitored by different sensor technology applications. Data can be provided to the worker by means of real-time feedback and access to data on demand. These feedback mechanisms may help workers to become aware of work exposures and encourages workers to act upon received feedback accordingly. Previous studies illustrated that sensor technology applications can be used to warn workers for occupational hazards, such as excessive heat strain [24] and support workers with mitigating incorrect work postures [25] and sitting behaviour [26]. Despite these initial positive results, more targeted research is needed to support workers using sensor technology applications in the workplace to measure and monitor their work exposures.

Many aspects might impede the implementation of sensor technology applications in the workplace, which should be taken into consideration for a successful implementation. Moreover, needs and preferences of workers regarding the use of sensor technology applications during work performance need to be identified. Therefore, in the present study we conducted a needs assessment among workers with physically demanding jobs to identify worker needs and preferences regarding the use of sensor technology applications in the workplace. We are particularly interested in (1) which work exposures workers would like to be measured and monitored, (2) the kind of sensor technology applications they would like to use, (3) which (pre)conditions need to be considered for measuring and monitoring in the workplace, (4) how to motivate workers for long-term use of sensor technology applications, and (5) which type of feedback workers would like to receive.

## Methods

### Design

Four on-site focus group sessions were conducted within four different companies. A qualitative study design was chosen, because in depth knowledge is lacking. The study protocol was approved by the Medical Ethics Board of the University Medical Center of Groningen. Anonymity, confidentiality, and the right to withdraw from the study at all times were guaranteed. We have used the COREQ checklist in reporting the methods used in our study (Additional file 1: COREQ checklist).

### Recruitment and inclusion criteria

Our sampling was based upon voluntary participation of workers with physically demanding work. Thirty workers with physically demanding work which were able to communicate in Dutch were included. Recruitment was conducted among four companies in the Northern part of the Netherlands, including participants from one small sized company (< 500 workers) active in civil engineering (C1), one medium sized company (≥ 500 workers) active in industrial cleaning (C2), and two large sized companies (≥ 1000 workers) active within the petrol industry (C3) and technical services (C4). The occupational health physician of the participating companies contacted supervisors of departments in which physically demanding work is performed and health problems are reported. Supervisors contacted workers within their department to participate in the study. Interested workers received an information letter and invitation to the focus group session at the workplace.

### Focus group interviews

The four focus groups were conducted in 2014; one at each company at the end of the workday. Each focus group met once and the focus group discussions lasted up to 1.5 h. The meetings were led by one researcher [WK], accompanied by a research assistant to take notes and to record the discussions.

A semi-structured interview schedule was used, facilitating wide-ranged explorations of the participants’ thoughts and experiences. All sessions started with a short introduction of a company representative to emphasize the importance of participation in this project, who left before the start of the focus group session. The use of sensor technology applications promoting health and wellbeing was introduced by a short video. During the meeting, five open questions regarding the use of sensor technology were discussed: (1) “Which work exposures would you like to see measured with sensor technology?”, (2) “What type of sensor technology applications would you like to use?”, (3) “What preconditions are necessary for use of sensor technology applications in the workplace?”, (4) “What motivates you for prolonged use of sensor technology applications?”, and (5) “How would you like to receive feedback on work exposures?”

### Data analysis

The focus group sessions were audio-recorded and transcribed verbatim. Transcripts were uploaded within Atlas.ti 7.5.10 (ATLAS.ti Scientific Software Development GmbH, Germany) for the coding process. To hierarchically structure encodings for analysis, Microsoft Excel was used. A content analysis was performed with an inductive approach [27, 28] by the first and second author [SS and WK]. All transcripts were coded by both researchers individually. Relevant passages were coded by adopting an open coding strategy. Assigned codes were compared and discussed until consensus was reached. The last author [SB] was consulted when agreement could not be reached. After all transcripts were coded and discussed, the first author [SS] structured the emergent themes hierarchically. Assigned codes were regrouped until agreement between the first author [SS] and second author [WK] was reached. Multiple steps were undertaken to assure research quality. These included (1) investigator triangulation via reflection on the research process, (2) peer review via discussions with the research team, and (3) enhancing confirmability by coding the transcripts by two investigators [SS, WK] and discussions on the performed data analysis.

## Results

### Sample characteristics

Thirty male workers with a mean age of 44.9 years (SD = 11.7) participated in the focus groups. Most workers were medium (58.6%) (i.e. intermediate vocal education, intermediate technical school) or low educated (34.5%) (i.e. lower general secondary education, lower technical school). On average, workers were employed for 14.9 years (SD = 12.3) within their company and reported an average work week of 41.9 h (SD = 3.5). Physically demanding work activities among our sample comprised industrial cleaning of machines and materials, excavation work and assembling and disassembling rig sites. Three of the four focus group sessions consisted of five to eight participants and one focus group consisted of 12 participants.

### Focus group outcomes

**Fig. 1:**
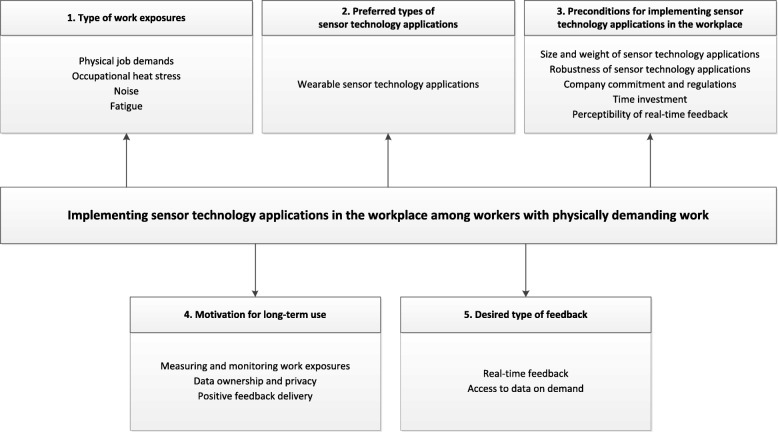
Thematic overview of reported needs and preferences for implementing sensor technology applications in the workplace among workers with physically demanding work

In our analysis, we distinguished the following themes; (1) work exposures workers would like to be measured, (2) the kind of sensor technology applications workers are interested in, (3) preconditions for measuring and monitoring work exposures with sensor technology applications in the workplace, (4) motivational aspects for long-term use of sensor technology applications, and (5) type of feedback workers would like to receive (see Fig. 1). The contents of each category will be elaborated below.

### Type of work exposures

Participants reported three work exposures (physical job demands, occupational heat stress, noise) and one health consequence (fatigue) which they would like to measure and monitor with sensor technology applications.

#### Physical job demands

Participants perceived high physical job demands, i.e. lifting heavy objects, pushing and pulling, working in awkward postures, working above head level and prolonged sitting. Subsequently, musculoskeletal pain is experienced at the end of the workday. *“You are digging all day long. At the end of the day your feet hurt, your hands hurt, everything hurts” (C1).* Participants considered the implementation of sensor technology applications in the workplace beneficial to receive warning signals when adopting incorrect postures or physical limits are reached. *“I am like, this device should tell me when I use my body incorrectly, so to speak” (C3).*

#### Occupational heat stress

Occupational heat stress is defined as *“the net load to which a worker is exposed from the combined contributions of metabolic heat, environmental factors, and clothing worn which results in an increase in heat storage in the body”* [29]. Participants experienced occupational heat stress from (1) performing strenuous physical activities in protective occupational clothing, (2) machinery which raises indoor temperature levels, and (3) exposure to sun and high outdoor temperatures in the summer. Participants wanted to become more aware of experienced occupational heat stress and whether and when acceptable health limits are exceeded. *“When you work in the sun, your body temperature increases. But what is the limit?” (C1).*

#### Noise

Participants mentioned that they are continuously working in noisy environments when they perform their daily work activities. *“That is the nature of our job of course. You are always surrounded by noise” (C4).* Some participants are aware that development of ear damage is a consequence of daily exposure to noise in their work. *“Anonymous group reports highlight that noise and deafness are one of these aspects of our job” (C2).* Participants were interested in whether their ear protection sufficiently protects their hearing and mentioned that they want to use sensor technology applications to receive warning signals when they are exposed to excessive noise levels. *“Look, I’ve got a device to measure the amount of H*_*2*_*S, which is considered as an occupational hazard. You receive devices to monitor such hazards, but not to monitor vibrations or noise for example. You can easily use a device which beeps or provides a light signal when you are exposed to too much noise” (C4).*

#### Fatigue

Fatigue was explicitly mentioned as a health consequence, impeding participants’ health and wellbeing. Participants considered fatigue as a risk factor for their personal safety when commuting and experience problems as a result of accumulated fatigue at work in their private life. *“Sometimes you are fatigued to such an extent that when you are in your car you think like hey, I really need to keep my focus” (C3).* And: *“Nowadays I eat with my family and go to bed at 21:30 because I am exhausted. And when I am back at work around 07:00, I am almost completely exhausted again at 13:00” (C1).*

### Preferred types of sensor technology applications

#### Wearable sensor technology applications

When reflecting on what type of sensor technology applications participants would like to use, a clear preference for wearable solutions was reported. To target physical job demands, participants suggested to adopt smart clothing or sensor suits to monitor posture. *“For instance, you could use a shirt to measure back posture as you stand incorrectly all day long. This shirt tells you to discontinue working in this posture and adopt another posture” (C1).* To prevent hearing loss, wearable noise detectors were suggested by participants which provide feedback on noise severity. *“Some kind of secondary pager which indicates that you are in a zone in which noise levels can be dangerous for your health” (C4).* Participants were not aware of the occupational heat stress they are exposed to, and wanted a device which is able to provide reliable information. *“We want to prevent occupational heat stress, but we cannot measure it, because we only can measure skin temperature” (C2)*. To prevent drowsy driving, participants mentioned a tool which provides a signal which indicates whether it is safe to drive home after work. *“That you receive a signal which advises you whether it is safe to drive. Or that you receive a warning signal, telling you to keep your focus” (C3)*.

### Preconditions for implementing sensor technology in the workplace

Participants reported five preconditions when implementing sensor technology in the workplace: size and weight of sensor technology applications, robustness of sensor technology applications, company commitment and regulations, obtrusiveness and perceptibility of feedback in the workplace.

#### Size and weight of sensor technology applications

Participants notified that the dimensions and weight of sensor technology applications can be potential barriers for use in the workplace. Sensor technology products should not impair performance of daily work activities. *“It should not limit your actions” (C3).* Furthermore, sensor technology products should not provide a physical burden when used. *“If you give us something which is not heavy and not difficult to use, then it is ok by me” (C1)*.

#### Robustness of sensor technology applications

The robustness of sensor technology applications is considered as one of the most important aspects impeding implementation in the workplace and includes resistance to water, lubricants, radiation, shock, dirt, shock, and fire. *“It must be water resistant, at least for some time, and dirt proof. Because I have to wear this device, it must be suitable for any given situation” (C1).*

#### Company commitment and regulations

Company commitment and worksite regulations have to be considered before implementing sensor technology applications. Participants reported for example that mobile phone use is not always authorized. *“Look, I am just not allowed to take a mobile phone with me on site” (C4).* Also the sensor technology applications should meet with (local) company standards. *“At certain locations you have to wear a helmet and clothing must be reflecting. When you meet those criteria, everything is possible. It should meet the standards of your employer” (C1).*

#### Time investment

Participants wanted to use sensor technology applications, which require a minimum amount of time investment. *“If you have to configure the device 10 times a day for example, I think it will get on my nerves” (C2).* And: *“Personally, I don’t want to spend time on data entry” (C1).* A clear instruction on how to use sensor technology applications within daily practice is essential to minimize necessary time investment. *“For me it’s like, well, I have no idea how it works and a ‘just measure it’ attitude doesn’t work for me. What do you have to wear? How does it work? What do I have to do?” (C1).*

#### Perceptibility of real-time feedback

Participants expressed that sensor technology applications should be used to provide perceptible awareness by real-time feedback signals or warning signals in hazardous conditions. *“It seems very easy to me to wear a bracelet which vibrates when you do something wrong, you will always perceive it” (C2)*. And: *“A device which warns you when noise levels are getting too high” (C4)*. However, work conditions can be challenging and provided real-time feedback should be suitable within this context. *“Our daily work activities in protective clothing include safety goggles. Wherever we are, we can get feedback directly when something is too heavy by a red flashing light. You immediately see what it is” (C2).*

### Motivation for long-term use

Three aspects were reported to facilitate or impede motivation for prolonged use of sensor technology applications: (1) Measuring and monitoring work exposures, (2) data ownership and privacy, and (3) positive feedback delivery.

#### Measuring and monitoring work exposures

To prevent injury and illness, participants wanted to become aware of the impact of work activities and work exposures on their health to take countermeasures when possible. *“Identifying physical load for example, and what I can do to improve this” (C3).* And: *“It is good to be aware about your health status. If they tell me that something is wrong, I’d rather know it now than over a year or two when you can’t do anything about it anymore” (C1)*. Real-time feedback was considered as an important aspect, helping participants to promote their health and wellbeing directly at the workplace. *“I think it (feedback on health) provides additional value, the ability to make it tangible” (C4)*.

#### Data ownership and privacy

Participants indicated that they want to obtain ownership over the collected data and decide with whom data will be shared. *“Give the data to the workers, then they can give it to the persons who want it or request it” (C2).* Furthermore, participants indicated that data sharing is possible. For example, general practitioners (GPs) would be allowed to review personal health data of the workers *“ When I declare that my GP can receive my results after performing a medical check, that is fine by me ” (C4)*. Participants acknowledged the expertise of internal health and safety services as well, but considered this department as unreliable due to possible conflicts of interest. The employer may use collected data to improve participants’ wellbeing in the work environment, but only when all personal information has been removed. *“I believe that if you make it work related, like lifting, it can be accessible for the employer. Things like heart rate are a little bit more personal” (C2).*

#### Positive feedback delivery

For a sustainable use of sensor technology applications, participants considered positive feedback to be essential. *“I don’t want to hear ‘you must do this with your back or you are not allowed to kick or jump on this’ daily” (C3).* Feedback is valued when it targets prevention of hazardous situations and contributes to improvements of health behaviour. *“That you receive a signal which tells you that it would be better to lift weights more with your legs” (C2).* Participants considered the interpretability of the results essential to identify which work exposures impede their health and wellbeing. *“So you can evaluate what is of influence (on your health) and in which way” (C3).* And: *“So we will receive specific values? I do not have the knowledge to interpret the results. If my heart rate increases, is it too high or is it just normal? And what about other values?” (C1).*

### Desired type of feedback

Participants wanted to receive real-time feedback on work exposures when their health and wellbeing is at stake and like to evaluate collected data to assess the extent in which work exposures affect their health and wellbeing.

#### Real-time feedback

Receiving real-time feedback in the workplace was desired by participants to become aware of any negative impact from work exposures on their health and wellbeing. Both tactile and visual feedback methods were considered suitable to provide real-time feedback in demanding work conditions. *“When you work in the factory, you have to lift pressure washer hoses over a balcony. When you reach your limit, a light should be blinking on your glasses, telling you to work differently or to work together with a colleague for example” (C2).* And: *“When you are handling heavy materials, it all vibrates. In those conditions, you won’t experience a (tactile) feedback signal” (C2).*

#### Access to data on demand

Participants reported a specific preference to use collected data to evaluate the impact of work exposures on their health and wellbeing. *“That you are capable of monitoring which factors are of influence (on your health) and to which extent” (C3)*. A specific preference for reports with quantitative data was mentioned by the participants to analyse the impact of work exposures, followed by advice to improve participants’ health and wellbeing in- and outside the workplace. *“Afterwards (after the measurement period), you create a report which demonstrates where things went wrong. That suits me best” (C1).* And: *“When I see numbers, it doesn’t tell me anything. But when I do a medical check and again after a few years and see that values have decreased by 50%, it makes me think, it makes me aware that I have to change” (C4).* Some participants mentioned that they prefer to discuss their results with health and safety experts and would like to have a follow-up with training or instruction sessions to prevent injury and illness in the future. *“Maybe the solution is also in good education and discussing what goes wrong, because often it is unintentional” (C2).*

## Discussion

This study provides insight in the needs and preferences of workers with physically demanding work regarding the implementation of sensor technology applications for measurement of exposures at the workplace. Participants reported three work exposures (physical job demands, occupational heat stress, noise) and one adverse health outcome (fatigue), which they would like to be measured with sensor technology applications. Sensor technology applications integrated in clothing or wearable devices were preferred by the participants to measure and monitor the dose and duration of work exposures. Data should be accessible on demand, but also real-time feedback should be provided by sensor technology applications when work exposures exhibit a significant hazard for worker health and wellbeing. For implementation in the workplace, sensor technology applications need to function safely in demanding work environments, need to comply with existing company health and safety regulations, and need to be convenient. To support the long-term use of sensor technology applications in the workplace, participants indicated a need to govern autonomy over their own data. Participants preferred to receive positive feedback from sensor technology applications when they actively try to reduce and prevent the negative impact of work exposures on their health and wellbeing. Moreover, participants wanted to share data to communicate with health professionals and health and safety experts, but expressed concerns about sharing data with their employer.

The majority of the participants indicated that they were aware of their exposure to different occupational hazards in the workplace, but experienced difficulties in assessing whether and when these work exposures may impact their health and wellbeing. Becoming aware of actual work exposures can be considered as a pivotal step to reduce or prevent future health problems. In behavioural change models such as the transtheoretical model of behavioural change [30], feedback has a prominent role to assist people in advancing throughout the different stages of behavioural change by raising awareness on actual health behaviour. As sensor technology applications are capable of continuously informing workers about work exposures and unhealthy behaviour in the workplace, workers may become more aware of the adverse effects of work exposures. Previous studies showed that increased awareness of work exposures or unhealthy behaviour helped workers to improve their work postures [25, 31], reduce their sedentary time [32], and increase their physical activity levels [33].

To raise awareness concerning adverse work exposures and facilitate appropriate action, different feedback mechanisms can be used, for example real-time feedback and access to data on demand. Participants particularly emphasised a need for wearable applications, which provide the ability to access data on demand and generate real-time feedback to warn them about poor work posture, high physical loads, occupational heat stress and excessive noise levels. Some sensor technology applications that meet these criteria have been developed and occasionally been implemented in the workplace to promote worker health and safety. For example, activity trackers have been used to support workers to analyse adopted work postures which can result into low back pain [34, 35], ear plugs have been developed to monitor core body temperatures to prevent heat stress [36] and sensors have been integrated in cars to detect lane deviations and eye movements to warn drivers for drowsy driving [37, 38]. Despite the potential of these innovations, implementation of sensor technology applications in the workplace so far remains limited.

Participants indicated that quality, comfort, and perceived ease of use may facilitate the use of sensor technology applications in the workplace. However, they also reported not to use those applications when they lack robustness to function properly in the workplace, are uncomfortable to wear, require too much time investments or are difficult to operate. The reported needs and preferences are in line with human-centered design principles for sensor technology applications to facilitate user acceptance, satisfaction and engagement [39]. With respect to the preferred wearable devices and smart garments, wearability may be an essential principle, which is considered a key factor for user satisfaction and engagement [39]. Furthermore, impaired wearability should also be avoided from a physical perspective. One study showed that problems with wearability may have caused perceived discomfort in the lower back [40], which may inflict future low back pain [41, 42]. Similar human-centered design principles are reflected in earlier studies on implementing sensor technology applications in healthcare settings. Within these domains, sensor technology applications are mainly used for remote health monitoring to alleviate pressure on healthcare services and nursing homes [43]. To facilitate remote healthcare monitoring, sensor technology applications are required to operate in daily living environments, which imposes demands on durability, reliability and robustness against external perturbations (e.g. shock or water resistant) [44]. Furthermore, a systematic review on demands for sensor technology applications among different patient groups identified that sensor technology applications should be compact, wearable, unobtrusive and easy to operate [45].

Concerns regarding data access and data privacy were also voiced by participants and may pose an additional barrier towards using sensor technology applications in the workplace. Participants indicated that they are open to sharing data with different stakeholders in- and outside the workplace when this may contribute towards their health and wellbeing, but also want to govern autonomy over their data to protect and manage access to their data. The need to protect and manage access to data has also been reported in earlier studies [46, 47], but it remains questionable whether workers are capable of taking this responsibility. Despite being aware of privacy infringements, people may still consider to disclose their data. This phenomenon is known as the privacy paradox [48] and the decision whether to disclose data or not is based on the privacy calculus theory, which comprises a comparison between associated benefits and drawbacks of data disclosure [49]. This decision can be difficult as people may be impaired to oversee the consequences of data disclosure due to an inability to thoroughly evaluate privacy risks and disclosure benefits [50]. In order to protect worker privacy, implementing sensor technology applications in the workplace needs to adhere to existing country specific legislation [51, 52] and workers should be provided with sufficient information to consider the positive and negative consequences of participation.

### Strengths and limitations

To our knowledge, this study is the first to identify specific needs and preferences regarding the use of sensor technology applications in the workplace from a worker perspective. Participants clearly indicated for which ends they wanted to use sensor technology applications in the workplace and which conditions should be met before advancing towards implementation. To reduce the probability of a selection bias, we deliberately organized our focus groups on-site at the end of a workday to minimize potential barriers towards participation and to encourage workers to participate in the focus group sessions as a team. This resulted in a sample of participants from all ages among a traditionally hard to reach audience which not only addressed the benefits, which is a strength of this study. However, as participants were recruited via open invitations among the companies and participation was voluntary, a certain selection bias cannot be excluded. The composition of our study design, the location and the sensitive topic may have introduced a potential bias which could influence the direction and magnitude of the study outcomes. Considering the variation within our study sample, this bias may be limited, but selective participation of early adopters which have an affinity with sensor technology applications may be overrepresented within our sample. A certain preference for sensor technology applications should be taken into consideration for future studies and adopting sensor technology applications within the workplace.

### Implications for research and practice

Our study illustrated that workers with physically demanding work are positive towards implementing sensor technology applications in the workplace, but specific reported needs and preferences should be considered in advance. Participants indicated that the use of sensor technology applications should primarily focus on work exposures. Specific preconditions and privacy concerns should also be taken into consideration. As needs and preferences may for example be subject to company, work activities or work environment, implementation of sensor technology applications require a tailored approach. A next step in research could be the development of tailored workplace based interventions on experienced work exposures with sensor technology applications, which uses the results of our study as a point of departure. For this purpose, the intervention mapping approach may be used. The intervention mapping approach was developed to assist researchers in developing, implementing and evaluating tailored interventions and includes a needs assessment as a fundamental step in this process [53].

## Conclusions

Sensor technology applications may support workers with physically demanding work to measure and monitor their work exposures. Potential barriers for implementation such as privacy aspects and quality, comfort and perceived ease of use of sensor technology applications need to be well considered to ensure successful implementation of sensor technology applications in the workplace.

## Additional file

Additional file 1 Consolidated criteria for reporting qualitative studies (COREQ): 32-item checklist. (DOCX 21 kb)

## Data Availability

The datasets used and/or analysed during the current study are available from the corresponding author on reasonable request.

## Abbreviations

C1 – C4: Focus group participant from one of the four participating companies
GP: General practitioner
